# Regulation of mRNA translation controls seed germination and is critical for seedling vigor

**DOI:** 10.3389/fpls.2015.00284

**Published:** 2015-04-28

**Authors:** Marc Galland, Loïc Rajjou

**Affiliations:** ^1^INRA, Institut Jean-Pierre Bourgin, UMR 1318 INRA/AgroParisTech, ERL Centre National de la Recherche Scientifique 3559, Laboratory of Excellence “Saclay Plant Sciences” (LabEx SPS)Versailles, France; ^2^Chair of Plant Physiology, AgroParisTechParis, France

**Keywords:** seed, plant, dormancy, germination, longevity, translation, mRNA, proteins

The control of seed germination capacity is a multi-level molecular process including, epigenetic, transcriptional, post-transcriptional, translational, and post-translational regulation (Rajjou et al., 2012). Since the beginning of the twenty-first century, a wide range of genetic, genomic, and post-genomic approaches have been used to decipher the underlying molecular and biochemical bases of dormancy, vigor, and longevity. In particular, the regulation of stored mRNA translation appears as an essential determinant of seed quality. Indeed, proteomic approaches unveiled the main importance of protein synthesis during seed germination (Rajjou et al., 2004; Kimura and Nambara, 2010), as well as factors involved in seed longevity (Rajjou et al., 2008). In aged seeds, the protein synthesis capacity decline together with the loss of germination potential. In contrast, both dormant and non-dormant Arabidopsis seeds display equal translational activity 1 day after imbibition although distinct protein pools are synthetized (Chibani et al., 2006). In sunflower, seed dormancy release by after-ripening would not be related to transcriptomic changes but associated with oxidation of specific subsets of stored mRNAs, thus impairing their translation (Bazin et al., 2011; Meimoun et al., 2014). In the aim to get a comprehensive view of translational control of seed dormancy and germination in sunflower, a microarray-based translatome analysis was performed, highlighting differential accumulation of polysome-associated mRNAs between dormant and non-dormant imbibed seeds (Layat et al., 2014). However, multiple ribosomes are not necessarily translationally active. Indeed, it has been observed that both active and stalled ribosomes are able to co-sediment during isolation of polysome complexes (Sivan et al., 2007). As a result, polysome profiling does not fully discriminate translationally active from repressed mRNAs. This concern should be particularly true in the case of dry seeds where polysomes would not be functional. Indeed, it has been observed that the ribosomes are condensed into regions consisting of closely packed particles in the dry seed related with a latent potential for protein synthesis (Chapman and Rieber, 1967). A rapid polysome formation occurs during early germination related with the transition from a dry and quiescent state to a fully imbibed and metabolically active state. In non-dormant Arabidopsis seeds, the comparison between the transcript changes and the protein changes from dry to 1d-imbibed seeds showed strong discordance (Galland et al., 2012). This is in accordance with previous work in plants reporting that the abundance of a transcript does not necessarily reflect its translation (Bailey-Serres et al., 2009). It is likely that when conditions are favorable for the maintenance of seed dormancy, translational selectivity will promote the translation of stored mRNA associated with maturation program (Arc et al., 2012). The time course of seed germination is related to both sequential and selective mRNA translation emphasizing a fine regulation of the translational machinery (Galland et al., 2014). Indeed, the temporal profiling of protein synthesis highlights that Arabidopsis seed germination consists of a series of sequential events overlapping with the three canonical phases of this process namely, water uptake (Phase I), lag phase (Phase II), and radicle growth (Phase III). Germination *sensu stricto* (i.e., prior to radicle emergence) refers to phases I and II while phase III consists of seedling growth resumption accompanied by both cell elongation and cell division. In the early step of water uptake, germination begins with a resumption of maturation program through the translation of mRNA associated with storage proteins and tolerance desiccation. This relates to an important checkpoint where, in a favorable environment, germination of non-dormant seeds is accompanied by a radical change in their translational program. Indeed, in the lag phase a sequential translation of mRNA related with antioxidant mechanisms, cell detoxification, protein fate, energy, and amino acids metabolism occurs. At the end of this lag phase, proteins involved in protein degradation and nitrogen remobilization are neosynthetized in preparation for the seedling growth. Therefore, it can be assumed that mRNA translation and protein post-translational modifications constitute the main levels of control for germination completion (Arc et al., 2011; Rajjou et al., 2012). These processes are highly regulated in plants and represent rapid and efficient way to cope with environmental variations. The regulation of mRNA translation is extremely complex and not explored enough in seed biology. Still the seed would be an excellent model for studying translation and selectivity mechanisms due to the presence of different mRNA populations in the dry mature seed. It seems relevant to conduct a comprehensive investigation about the mRNA recruitment by the nuclear cap-binding complex (CBC) and by the cytoplasmic mRNA CBC (eIF4F) since several phenotypes were observed in mutant seeds affected in genes involved in these mechanisms. The eIF4F and polyA-binding protein (PABP) promotes the transcript stabilization and the ribosome–mRNA interactions (Gingras et al., 1999; Hinnebusch and Lorsch, 2012). The exon junction complex (EJC) would be involved in plant translational selectivity since it links the different aspects of mRNA biogenesis, such as transcription, splicing, export, surveillance, and nonsense-mediated mRNA decay (NMD) (Nyikó et al., 2013). Particular attention should be paid to DEAD-box RNA helicases presumably involved in translation by assisting ribosome maturation (Cordin et al., 2006). Indeed, the Plant RNA helicase75 (PRH75) have been shown to be a target of the Protein Isoaspartyl Methyltransferase 1 (PIMT1) that repair non-functional isoAsp residues upon seed deterioration (Nayak et al., 2013). This is probably an explanatory element of the impairment of seed vigor and longevity in PIMT-deficient genotypes (Ogé et al., 2008; Verma et al., 2013). In addition, the cap-independent process through direct mRNA recruitment by ribosomal subunits on an internal ribosome entry sites (IRES) would be possible since mature seeds have proteins named ITAFs (IRES-specific cellular trans-acting factors) involved in this process (Catusse et al., 2008). To date, mRNA decay and translational repression by small RNAs remain non-addressed in seed biology but may be a determinant way for translational selectivity. The impact on translation of the plant TOR (target of rapamycin) protein kinases pathway is associated with abscisic acid (ABA) and growth processes in plants (Deprost et al., 2007). Further investigation of TOR-dependent phosphorylation signaling in seed dormancy, germination, and longevity appears required. The involvement of different ribosomal subunits and their post-translational regulation also remains unexplored in the control of seed germination. Thus, through this reasoning about the central role of translational regulation in the control of germination, future work on this issue should provide a better understanding of the mechanisms underlying seed physiology and provide robust markers for seed vigor.

## Conflict of interest statement

The authors declare that the research was conducted in the absence of any commercial or financial relationships that could be construed as a potential conflict of interest.
